# Editorial: Immunology of HPV Infection and Vaccination: Progress and Challenges

**DOI:** 10.3389/fimmu.2021.665463

**Published:** 2021-03-12

**Authors:** Paul V. Licciardi, Ian H. Frazer, Suzanne M. Garland, Kim Mulholland

**Affiliations:** ^1^ Infection and Immunity, Murdoch Children’s Research Institute, Melbourne, VIC, Australia; ^2^ Department of Paediatrics, University of Melbourne, Melbourne, VIC, Australia; ^3^ Diamantina Institute, The University of Queensland, Brisbane, QLD, Australia; ^4^ Department of Obstetrics and Gynecology, University of Melbourne, Melbourne, VIC, Australia; ^5^ Centre for Women’s Infectious Diseases Research, The Royal Women’s Hospital, Melbourne, VIC, Australia; ^6^ Department of Infectious Disease Epidemiology, London School of Hygiene and Tropical Medicine, London, United Kingdom

**Keywords:** human papillomavirus, vaccination, immune response, HPV infection, protection

## 

Human papillomavirus (HPV) infection affects both men and women and causes a wide range of diseases from benign lesions, such as genital warts, to more serious diseases such as anogenital and oropharyngeal cancers. HPV has evolved effective strategies to evade the host immune system and while antibodies are important, our understanding of other host immune factors involved in protection, particularly at the mucosal surface, is poorly understood. Prophylactic HPV vaccines are highly effective in preventing vaccine genotype-related-HPV infections and their associated diseases. While these vaccines do not clear existing lesions, there are several promising therapeutic vaccines targeting HPV-associated lesions that are undergoing clinical trials. A number of important questions related to HPV infection and vaccination remain unanswered. This includes identification of novel immunological markers of protection (including antibody, cellular and mucosal responses) against oncogenic and non-oncogenic HPV types, as well as understanding the critical immune responses required for optimal immune protection in high-risk groups who are particularly vulnerable to HPV infection and associated diseases.

This collection of articles under this Research Topic focuses on current concepts and state of the art knowledge on the immunology of HPV infection and vaccines.

The review article by Zhou et al. provides an overview of the current landscape of HPV vaccines, including important aspects such as immunogenicity, cross-protection and potential adverse reactions. They refer to the global call for action on the elimination of cervical cancer by WHO and highlight the current strategies needed to improve vaccine coverage in both high-income and low-income settings. Importantly, using China as an example where HPV disease burden has increased over the last 20 years, the recent licensure of prophylactic HPV vaccines is a fundamental step that will go a long way to reducing the prevalence of HPV-associated diseases among the Chinese population in future years.

Monitoring the effectiveness of HPV vaccines during epidemiological studies and clinical trials relies on accurate measurement of both HPV DNA, as well as HPV-specific neutralizing antibodies. While these are routinely assessed using swabs and blood, respectively, novel approaches such as urine have also been investigated. In their Perspective article, Pattyn et al. describe the benefits and accuracy of first-void urine for the measurement of both HPV DNA, as well as antibodies. This sample represents a non-invasive approach to measuring HPV DNA and particularly antibodies that are present at the cervicovaginal surface where HPV typically infects. They suggest that while there are limitations with this type of approach, HPV-specific antibodies can be detected with moderate to good correlations with serum antibodies, offering a promising alternative for monitoring vaccination programs post-implementation. Indeed, simpler approaches to measurement of HPV antibodies are needed as the ‘gold-standard’ pseudovirus (PsV)-based neutralization assays are labor intensive and require a high level of technical expertise while the virus-like particle (VLP)-based assays require specialized reagents and equipment which can be difficult to source. The manuscript by Toh et al. describes the use of a PsV ELISA to measure HPV-specific antibodies following vaccination, showing that the production of PsV compared with VLP is simpler and provided excellent correlations with the neutralization assay, offering an alternative method for HPV antibody measurement.

Understanding the immunological basis of protection against HPV infection is also critical in the context of developing and harnessing therapeutic strategies to improve survival. To this end, Zhang et al. reported that in mice, CD40 activation accelerated vaccine-induced HPV16 tumor-specific CD8+ Tscm (stem-like memory) formation which were more potent and longer-lived that other HPV-specific CD8+ T cells. Importantly, there was a higher proportion of Tscm cells in patients who were HPV16+ but tumor negative compared with HPV16+ cancer patients. This offers new insights into cancer immunotherapy strategies aimed at targeting these cells to improve patient outcomes. In another paper by Zottnick et al., they highlight the advantages of newly developed orthotopic models of HPV cancer using a humanized mouse system. Such an approach would overcome the limitations of existing preclinical mouse models and allow novel HPV therapeutic vaccination strategies to be examined for their ability to induce human immune responses at the site of infection and hopefully improve translation to the clinic.

Novel antiviral compounds that can disrupt HPV infection are also an attractive area of research. In their manuscript, Skeate et al. demonstrate using *in vitro* models that the theta-defensins from rhesus macaques have unique properties in terms of inhibiting HPV infection through preventing binding to specific cell surface receptor complexes on target epithelial cells. The development of low-cost antiviral strategies that prevents infection and transmission of high-risk HPV types would be an important component in the overall goal to reduce HPV infection and their associated diseases globally.

This Research Topic provides an up-to-date summary of the latest research and insights into the immunology of HPV infection and vaccination that will ultimately provide the basis for improved strategies to reduce HPV-associated diseases in the future.

## Author Contributions

PVL drafted the manuscript. All authors contributed to the article and approved the submitted version.

## Funding

PVL is supported by an Australian National Health and Medical Research Council Career Development Fellowship Level 2 (GNT1146198). IHF is supported by an Australian National Health and Medical Research Council Investigator Grant (GNT1173927). SMG is supported by an Australian National Health and Medical Research Council Investigator Grant (GNT1197951).

## Conflict of Interest

The authors declare that the research was conducted in the absence of any commercial or financial relationships that could be construed as a potential conflict of interest.

